# Long-term effects of aromatase inhibitors on body mass index among postmenopausal breast cancer survivors in Africa: observational cohort study

**DOI:** 10.1186/s13104-023-06301-6

**Published:** 2023-03-13

**Authors:** Jean Paul Muambangu Milambo, Peter S Nyasulu, John M Akudugu, James Ndirangu

**Affiliations:** 1 Department of Health Sciences, Division of Public Health, University of Free state, 205 Nelson Mandela Dr, Bloenfontein , 9301 South Africa; 2 Department of Global Health, Division of Epidemiology and Biostatistics, Faculty of Medicine and Health Sciences, Stellenbosch University, Stellenbosch, South Africa; 3 Division of Radiobiology, Department of Medical Imaging and Clinical Oncology, Faculty of Medicine and Health Sciences, Stellenbosch University, Stellenbosch, South Africa; 4 Department of Health Sciences, Division of Public Health, University of Free State, Bloenfontein , South Africa

**Keywords:** Aromatase inhibitors, High sensitivity C-reactive protein, Body mass index, Postmenopausal breast cancer, And multiple imputations

## Abstract

**Purpose:**

this study was conducted to assess the impact of AIs on body mass index and high sensitivity as prognostic predictors to be incorporated into point of care technology (POCT) testing in postmenopausal breast cancer women after a 24 month follow up in Africa. An observational cohort study was conducted; including 126 female BC patients with stages ranging from 0-III initially subjected to AIs and subsequently followed up for 24 months. Multiple imputation model was conducted to predict missing data.

**Results:**

Random effects model was used to monitor the changes over the time. The study revealed stronger statistically association between BMI and homocysteine (p = 0.021, 95%CI: 0.0083 to 0.1029). Weight and total body fat were strongly associated after 24 months follow up. Hs-CRP was associated with BMI (p = 0.0001), and hs-CRP was associated with other biomedical markers such as calcium (p = 0.021, 95% CI: 0.01 to 0.10), phosphate (p = 0.039, 95%CI: 0.01 to 0.10), and ferritin (p = 0.002, 95%CI: 0.02 to 0.08) and calcium. The patients subjected to AIs are likely to develop cardiovascular adverse events. POCT of care strategy which include clinical, biomedical and genetic predictor’s measurement is required to improve BC survivorship.

**Supplementary Information:**

The online version contains supplementary material available at 10.1186/s13104-023-06301-6.

## Introduction

Obesity and mediators of inflammation have been identified as the most important risk and predictive factors in postmenopausal breast cancer survivors using aromatase inhibitors (AIs) [1]. According to Bardia et al., (2012), the 10-year predicted recurrence risk for cardiovascular disease (CVD) equals or exceeds that of BC in postmenopausal women [2]. CVD-related endocrine therapies impact on health-related quality of life (HRQOL) in postmenopausal BCS [3–9]. Maintaining patient adherence is a critical situation experienced among BCS subjected to long-term endocrine therapies [10]. Failure to follow-up and missing data in cancer survivors is well acknowledged; and as a result, there is increased morbidity and mortality [11]. Given that the missing data containing known and unknown reasons are common in cancer survivors, using historical patient information stored in clinical databases is currently applied in computational science to predict patient outcomes using modeling approaches [12–15]. Common retrospective data collection platforms are characterized by incomplete useful data, and these challenges may lead to limitations in statistical analyses of patient prognoses [16]. Researchers have recommended different statistic approaches, including advantages and disadvantages in order to estimate patient outcomes using mathematical modeling based on assumptions of both baseline data and literature [17]. Data is lacking on the effects of AIs on clinical markers (e.g. BMI) and inflammatory markers (e.g. hs-CRP) in postmenopausal survivorship as a result of BC treatment strategies in the African setting. Therefore, this study was conducted to assess the impact of AIs on hs-CRP and BMI, as prognostic markers to be incorporated in POCT in postmenopausal BCS in Africa over a 24-month follow-up.

## Main texts

### Methods and study design

A prospective observational cohort study non comparison group was conducted in this study. However, unlike a true experiment, a quasi-experiment does not rely on random assignment. Instead, subjects are assigned to groups based on non-random criteria. A longitudinal study can be used with different treatment strategies without control group. But the stratification was used in this study to minimize selection biases and effects of different treatment modalities. Breast cancer patients are likely to take different treatment strategies, using placebo is not ethically advised due to complexity of the breast cancer and associated comorbidities. This justify the absence of control group. Tygerberg Hospital is a tertiary hospital where many BC patients are referred from urban and rural primary care clinics for specialized BC surgery or radiation therapy. A prospective cohort study was performed in parallel with ongoing generation of a BC biobank and genomics database/registry developed under reference number N09/08/224. Inclusion and excluded criteria for postmenopausal women (aged 45–80 years) were documented following Stage 0-III ER-positive breast cancer, subjects with available data in genomics database and the National Health Laboratory Service (NHLS) were selected. Eligible patients were stratified based on BMI, hs-CRP, waist circumference and breast cancer strategies. Clinical and biomedical profiles of 126 BC patients fulfilled the eligibility criteria. The primary outcome was the multiple imputations of the clinical and biomedical survival outcomes based on the existing baseline data. The secondary outcome included development of a prediction model to examine the effects of AIs on BMI, hs-CRP, and other inflammatory markers after 24 months of AIs use based on the mean change of the above markers over time. Detailed of methodology and statistical analysis were published elsewhere [18]. This study was approved by Health by the HREC of Stellenbosch University [18]. Descriptive and analytical statistics were conducted as appropriate and STATA version 16 was used for analysis [18] and others published articles [19–23].

## Results

A convenience sample of 126 participants was considered for this analysis. Descriptive analysis was reported previously [24]. About 56 (44.44%) participants received more than 4 treatment options, 39 (30.95%) received at least 4 treatment options and 31 (24.60%) received less than 4 treatment options. The mean and standard variation by treatment options were provided. These including height, BMI, hs-CRP, weight, phosphate, homocysteine, ferritin, TBF and hip circumference. Table 1 provides the baseline characteristics of the postmenopausal breast cancer survivors. Random linear effects model revealed stronger statistically association between BMI and homocysteine (p = 0.021, 95%CI: 0.0083 to 0.1029). Weight and total body fat were strongly associated after 24 months follow up. In addition, hs-CRP was associated with BMI (p = 0.0001), and hs-CRP was associated with other biomedical markers such such as calcium (p = 0.021, 95% CI: 0.01 to 0.10), phosphate (p = 0.039, 95%CI: 0.01 to 0.10), and ferritin (p = 0.002, 95%CI: 0.02 to 0.08). There was statically significant correlation between cholesterol, BMI, phosphate, and hypertension after 24-month follow-up. Table 2 provides the outputs of the mean changes of inflammation markers at baseline and after month 24. The correlation between BMI, TBF, weight, hs-CRP, homocysteine, ferritin and calcium between baseline and after 24 months of follow-up. Hypertension was associated with BMI, weight and homocysteine after 24-month follow-up. Table 3 provides the effects of AIs on inflammatory markers at baseline and month 24th using multiple imputation model. Details of the analyses are published as PhD thesis on Website of Stellenbosch University. https://scholar.sun.ac.za*› bitstream › handle › m*.

**Table 1:** Baseline characteristics of the breast cancer patients

Parameters	Treatment 1	Treatment 2	Treatment 3	p-value
N	56	39	31	
BMI, mean (SD)	31.648214 (7.9894019)	31.130769 (6.8315119)	33.212903 (8.8297883)	0.53
Hs-CRP, median (IQR)	6.935 (2.295–12.62)	6.53 (2.98–13.29)	5.44 (2.22–12.41)	0.97
phosphate, median (IQR)	1.19 (1.09–1.295)	1.19 (1.03–1.32)	1.22 (1.12–1.31)	0.81
Calcium, mean (SD)	2.3473214 (0.09290251)	2.324359 (0.12069524)	2.356129 (0.09054198)	0.39
Phosphate, median (IQR)	4.9 (3.65–6.65)	5.4 (4.2–7.9)	5 (3.4–7.5)	0.23
Ferritin, median (IQR)	87 (34–145)	58 (35–137)	86 (34–156)	0.75
Homocysteine, median (IQR)	11.25 (9.7- 13.35)	11.6 (10- 15.3)	10.5 (9.2–14.1)	0.66
Total Body Fat, median (IQR)	48.75 (42- 53.3)	46 (43.8–52.2)	47.8 (43.5–51.5)	0.76
Hip Circumference, mean (SD)	111.86607 (16.88046)	111.11538 (14.125128)	119.91935 (14.208036)	0.035

*BMI = body mass index, pth = phosphate, SD = standard deviation, IQR = interquartile range, Hs-CRP = high sensitivity C-reactive protein. (1) = all types of surgeries, neo and adjuvant chemotherapy, radiation therapy, and aromatase inhibitors, (2) = radiation therapy, neo and adjuvant therapy, and aromatase inhibitors, and (3) = surgery combined with aromatase inhibitors only*

**Table 2:** The effects of AIs on BMI and hs-CRP after 24 months of follow-up

Aromatase inhibitors at month 24	Coef	p-value	95% CI
TBF	0.119	0.0001	0.070 to 0.160
Weight	0.353	0.001	0.340 to 0.360
Hs-CRP	0.006	0.113	-0.001 to 0.014
Homocysteine	0.055	0.021	0.008 to 0.102
Phosphate	13.256	0.056	-0.364 to 26.878
Calcium	-35.945	0.017	-65.530 to -6.359
Phosphate	0.928	0.039	0.0470 to 1.809
Ferritin	0.052	0.002	0.019 to 0.084
BMI	0.835636	0.001	0.502 to 1.168

Legends BMI = body mass index, coef = coefficient, pth = phosphate, hs CRP = high sensitivity C reactive protein

**Table 3:** Baseline characteristics of the breast cancer patients

Parameters	Treatment 1	Treatment 2	Treatment 3	p-value
N	56	39	31	
BMI, mean (SD)	31.648214 (7.9894019)	31.130769 (6.8315119)	33.212903 (8.8297883)	0.53
Hs-CRP, median (IQR)	6.935 (2.295–12.62)	6.53 (2.98–13.29)	5.44 (2.22–12.41)	0.97
phosphate, median (IQR)	1.19 (1.09–1.295)	1.19 (1.03–1.32)	1.22 (1.12–1.31)	0.81
Calcium, mean (SD)	2.3473214 (.09290251)	2.324359 (.12069524)	2.356129 (.09054198)	0.39
Phosphate, median (IQR)	4.9 (3.65–6.65)	5.4 (4.2–7.9)	5 (3.4–7.5)	0.23
Ferritin, median (IQR)	87 (34–145)	58 (35–137)	86 (34–156)	0.75
Homocysteine, median (IQR)	11.25 (9.7-13.35)	11.6 (10-15.3)	10.5 (9.2–14.1)	0.66
Total Body Fat, median (IQR)	48.75 (42-53.3)	46 (43.8–52.2)	47.8 (43.5–51.5)	0.76
Hip Circumference, mean (SD)	111.86607 (16.88046)	111.11538 (14.125128)	119.91935 (14.208036)	0.035

*BMI = body mass index, pth = phosphate, SD = standard deviation, IQR = interquartile range, Hs-CRP = high sensitivity C-reactive protein. (1) = all types of surgeries, neo and adjuvant chemotherapy, radiation therapy, and aromatase inhibitors, (2) = radiation therapy, neo and adjuvant therapy, and aromatase inhibitors, and (3) = surgery combined with aromatase inhibitors only.*

## Discussion

This study have contributed to external validity of the findings reported in literature [24]. The study revealed a stronger statistical association between BMI and homocysteine, while weight and total body fat were strongly associated after the 24-month follow-up. Hs-CRP was associated with BMI (p = 0.0001), and hs-CRP was associated with other inflammatory markers such as calcium, phosphate, and ferritin.

These findings are supported in literature [25, 26]. In addition, the mean changes after 24 months of baseline BMI, hs-CRP and estimated BMI and hs-CRP at month 24 were not statistically significant. Lack of statistically significant changes may be related to the small sample size used to build these models [27]. The findings of the present modeling are supported by a Women’s Health Study in which 27,919 postmenopausal women were followed-up for 10 years [11]. In this large study, a total of 892 women developed invasive breast cancer [7]. Moreover, a study conducted to assess the effects of AIs on CVD adverse events occurrence during a one-year follow-up in postmenopausal BCS showed no significant changes on hs-CRP, cholesterol levels, and blood pressure observed between intervals versus control groups. However, the controversy was identified in other study after 5 years of AIs therapy [28]. Clinicians should consider referring the highest risk patients for careful clinical and biochemical assessment to prevent long-term adverse events [7, 29].

The association between BC and hs-CRP are document including different biomedical and genetic pathways [14, 30–33]. This may include cardiovascular toxicity due to inhibition of CYP19A1 [34, 35] as well as the link between hs-CRP and AIs [36, 37].

This model is simple, easy to interpret, scientifically acceptable, and widely available [16, 17, 38]. However, it is important to note that real world events may not correspond with the mathematical assumptions of a linear model. In this case, the research team used real patient data for inference and published studies in other settings for model validation. Multiple approaches such as Bayesian Networks (BNs), machine computational technologies are approved in cancer studies to predict survivorship parameters for their accuracy in predictive models [16]. The findings from this study will be shared with clinicians and an additional assessment, using a large sample and population diversity will be proceeded for external validity.

## Conclusion

This study showed a correlation between BMI, TBF, weight, hs-CRP, homocysteine, ferritin and calcium between baseline and after 24 months of follow-up. Hypertension was associated with BMI, weight and homocysteine after 24-month follow-up. Routine assessment of hs-CRP and BMI are identified independent prognostic markers of CVD related adverse events in postmenopausal breast cancer survivors using AIs. Further studies on implementation of point-of-care testing incorporating clinical and biomedical markers are needed to predict AIs-associated adverse events in postmenopausal breast cancer survivors in different African settings.

## Limitations and recommendations

The analysis conducted in this study was focused only on BMI and hs-CRP as predictors of CVD risk factors in postmenopausal BCS, using baseline data from main study. Other determinants of survivorship in cancer patients [23], such as types of treatment, reasons for loss to follow-up and hazard survival curve analysis [39]. These results may be affected by the small sample size and convenience sampling technique used in this study. The correlation between clinical, biochemistry and genomic predictors should be performed in a larger prospective study using a number of genes already published in literature [40, 41]. Since the number of genetic predictors may be much larger than clinical or biomedical markers, model comparisons should be performed to confirm the results of this study.

## Electronic supplementary material

Below is the link to the electronic supplementary material.

Supplementary Material 1

## Data Availability

Not applicable.

## Abbreviations

AIs: aromatase inhibitors
ANNs: Artificial Neural Networks
CVD: cardiovascular diseases
BMI: body mass index
BCS: breast cancer survivors
BC: breast cancer
Hs-CRP: high sensitivity C-reactive protein
PMM: Predictive Mean Matching
TBF: Total body fat
