# Supplementary material for: Long-term effects of aromatase inhibitors on body mass index among postmenopausal breast cancer survivors in Africa: observational cohort study
Source: BMC Res Notes. 2023 Mar 13;16:37. doi: 10.1186/s13104-023-06301-6 (PMC10012500; doi:10.1186/s13104-023-06301-6)
Supplement: Supplementary file 1 — Supplementary Material 1 [file 13104_2023_6301_MOESM1_ESM.docx]

**Table 3: Correlation between comorbidities and inflammatory markers in postmenopausal breast cancer patients**

|  | |  | | **Hypertension/CVD** | | |  |
| --- | --- | --- | --- | --- | --- | --- | --- |
| **Correlation** | | **Total** | | **No** | | **Yes** | **p-value** |
|  | | N=126 | | N=50 | | N=76 |  |
| BMI | | 31.8 (7.8) | | 28.8 (6.9) | | 33.7 (7.8) | <0.001 |
| Ferritin | | 85 (35-150) | | 60 (32-123.5) | | 98 (44-158) | 0.081 |
| Homocysteine | | 11.3 (9.4-13.4) | | 11 (9-13.3) | | 11.4 (9.9-13.9) | 0.30 |
| Hs-crp | | 5.52 (2.3-12.33) | | 4.07 (1.73-10.48) | | 7.4 (2.845-13.4) | 0.064 |
| Phosphate | | 1.2 (1.09-1.3) | | 1.19 (1.07-1.2) | | 1.2 (1.09-1.36) | 0.35 |
| TBF | | 45.5 (6.0) | | 44. 2(5.9) | | 46.3 (5.9) | 0.083 |
| Calcium | | 2.3 (2.2-2.4) | | 2.33 (2.2-2.3) | | 2.3 (2.2-2.4) | 0.079 |
|  |  | | **Hot Flashes** | | | |  |
|  | Total | | No | | Yes | | p-value |
|  | N=124 | | N=46 | | N=78 | |  |
| BMI | 31.8 (7.40) | | 32.49 (7.4) | | 31.47 (7.4) | | 0,46 |
| Ferritin | 105.2 (97.6-112.5) | | 103.2 (98.62-115.7) | | 106.2 (97.6-110.9) | | 0,48 |
| Homocysteine | 11.9 (10.7-13.4) | | 11.2 (10.7-12.8) | | 12.2 (10.8-13.9) | | 0,09 |
| Hs-crp | 12.7 (7.5-18.7) | | 12.7 (9.6-18.7) | | 12.7 (6.2-19.1) | | 0,61 |
| Phosphate | 1.2 (1.17-1.2) | | 1.2 (1.17-1.2) | | 1.2 (1.17-1.2) | | 0,55 |
| TBF | 46.5 (4.0) | | 46. 9(3.8) | | 46.3 (4.1) | | 0,37 |
| Calcium | 2.34 (2.34-2.35) | | 2.35 (2.34-2.35) | | 2.34 (2.33-2.35) | | 0,25 |
|  | |  | | **Cholestérol** | | |  |
|  | | Total | | No | | Yes | p-value |
|  | | N=126 | | N=87 | | N=39 |  |
| BMI | | 31.8 (7.3) | | 32.2 (7.04) | | 31.07 (8.03) | 0.42 |
| Ferritin | | 104.7 (97.6-112.4) | | 106.4 (97.6-113.5) | | 102.6 (97.1-110.1) | 0.35 |
| Homocysteine | | 12.0 (10.7-13.4) | | 11.6 (10.6-12.8) | | 12.3 (10.8-14.0) | 0.048 |
| Hs-crp | | 12.7 (7.5-18.7) | | 13.8 (7.3-20.5) | | 11.6 (7.5-17.5) | 0.19 |
| Phosphate | | 1.2 (1.1-1.2) | | 1.2 (1.17-1.2) | | 1.2 (1.1-1.2) | 0.036 |
| TBF | | 46.5 (4.0) | | 46.8 (3.8) | | 46.0 (4.4) | 0.31 |
| Calcium | | 2.3 (2.34-2.3) | | 2.34 (2.3-2.3) | | 2.3 (2.3-2.3) | 0.87 |
|  | |  | | **Diabetics** | | |  |
|  | | Total | | No | | Yes | p-value |
|  | | N=126 | | N=108 | | N=18 |  |
| BMI | | 31.8 (7.3) | | 31.7 (7.5) | | 32.4 (6.5) | 0,72 |
| Ferritin | | 104.7 (97.6-112.4) | | 105.4 (97.6-112.5) | | 103.9 (99.3-112.2) | 0,87 |
| Homocysteine | | 12.0 (10.7-13.4) | | 12.0 (10.7-13.4) | | 11.2 (10.6-13.9) | 0,41 |
| Hs-crp | | 12. 7(7.5-18.7) | | 12.6 (7.4-18.7) | | 12.8 (11.8-19.1) | 0,47 |
| Phosphate | | 1.2 (1.1-1.2) | | 1.2 (1.17-1.2) | | 1.2 (1.1-1.2) | 0,71 |
| TBF | | 46.5 (4.0) | | 46.5 (4.1) | | 46. 7(3.2) | 0,85 |
| Calcium | | 2.3 (2.3-2.3) | | 2.3 (2.3-2.3) | | 2.3 (2.3-2.3) | 0,89 |

*No= the patients who did not complete the study follow-up, Yes= patients who completed the prescribed therapy.*
